# Digital Determinants of Health: Health data poverty amplifies existing health disparities—A scoping review

**DOI:** 10.1371/journal.pdig.0000313

**Published:** 2023-10-12

**Authors:** Kenneth Eugene Paik, Rachel Hicklen, Fred Kaggwa, Corinna Victoria Puyat, Luis Filipe Nakayama, Bradley Ashley Ong, Jeremey N. I. Shropshire, Cleva Villanueva

**Affiliations:** 1 MIT Critical Data, Massachusetts Institute of Technology, Cambridge, Massachusetts, United States of America; 2 Laboratory for Computational Physiology, Massachusetts Institute of Technology, Cambridge, Massachusetts, United States of America; 3 Research Medical Library, MD Anderson Cancer Center, Houston, Texas, United States of America; 4 Department of Computer Science, Mbarara University of Science & Technology, Mbarara, Uganda; 5 University of the Philippines College of Medicine, Manila, Philippines; 6 Department of Ophthalmology, São Paulo Federal University, São Paulo, Brazil; 7 Department of Neurology, Neurological Institute, Cleveland Clinic, Cleveland, Ohio, United States of America; 8 Morehouse School of Medicine, Atlanta, Georgia, United States of America; 9 Instituto Politécnico Nacional, Escuela Superior de Medicina, Mexico City, Mexico; St Luke’s Medical Center, PHILIPPINES

## Abstract

Artificial intelligence (AI) and machine learning (ML) have an immense potential to transform healthcare as already demonstrated in various medical specialties. This scoping review focuses on the factors that influence health data poverty, by conducting a literature review, analysis, and appraisal of results. Health data poverty is often an unseen factor which leads to perpetuating or exacerbating health disparities. Improvements or failures in addressing health data poverty will directly impact the effectiveness of AI/ML systems. The potential causes are complex and may enter anywhere along the development process. The initial results highlighted studies with common themes of health disparities (72%), AL/ML bias (28%) and biases in input data (18%). To properly evaluate disparities that exist we recommend a strengthened effort to generate unbiased equitable data, improved understanding of the limitations of AI/ML tools, and rigorous regulation with continuous monitoring of the clinical outcomes of deployed tools.

## Author summary

New technologies and tools for Artificial intelligence (AI) and machine learning (ML) in healthcare are continually advancing, leading to new digital tools that can improve the delivery of care. However, as these computer-based tools improve, they become more complicated and less transparent. These tools use gathered data from medical practice or clinical outcomes to build mathematical models to make recommendations that assist clinicians to treat patients. Unfortunately, when the data going in is biased, then the digital tools themselves are corrupted to perpetuate or even amplify the health disparities, leading to worsened inequity against already vulnerable populations. Data poverty describes when certain people groups are underrepresented in generated health data, so that they may actually be harmed by these new tools. Our review looks at the established state of research into health data poverty. We attempt to characterize the scope and findings of these papers, assess the challenges within the field, and draw some recommendations on how to begin to approach the difficult problem of health data poverty.

## Introduction

While many laud the potential for technology improving the quality and delivery of healthcare, we must be vigilant to avoid exacerbating existing health disparities [1]. One area of focus toward addressing these inequalities is to resolve the expanding problem of health data poverty, defined as "the inability for individuals, groups, or populations to benefit from a discovery or innovation due to insufficient data that is adequately representative" [1]. Utilizing non-inclusive health data from underrepresented populations in clinical applications often leads to misapplied generalizations and worse outcomes [1]. Furthermore, continuing to build technologies based on marginalized datasets perpetuate or can even amplify disparities rather than mitigating them [2]. Despite technological advancements, communities with lower health outcomes often continue to have poorer outcomes regardless of the improvements in technology [3]. This disconnect is exemplified in the burgeoning application of artificial intelligence and machine learning (AI/ML) in healthcare, where studies have demonstrated that biases can be driven by discrepancies or gaps in the available healthcare data [4–6].

### The hope of AI/ML

Within digital health, advancements in AI/ML leverage computer-based mathematical models to analyze collected health data and predict outcomes [7]. These applications have an enormous potential to transform healthcare, leveraging vast amounts of health data and steering healthcare away from anecdotal medicine toward enhanced evidence-based care [8]. AI/ML may provide researchers and clinicians with additional tools necessary to administer high-performance medicine with greater efficiency, better workflow, and improved prediction of health outcomes [9–11] AI algorithms have been applied across a myriad of specialties, including radiology, pathology, dermatology, intensive care medicine, oncology, genetics, and ophthalmology [12–20]. Some algorithms, in controlled settings, have been shown to outperform trained clinicians in detecting pneumonia, breast cancer, age-related macular degeneration, and diabetic retinopathy [21–23]. While the algorithms and tools have been developing at a rapid pace, the true effectiveness and value of AI/ML depend directly on the quality of the input data [24].

### The pervasiveness of bias in healthcare

Bias is insidious and infiltrates healthcare delivery often without realization. While most clinicians attempt to avoid explicit biases, implicit biases are introduced unconsciously and systemic biases are embedded within our institutions and systems [25–27]. Understanding the presence of bias throughout healthcare is critical to addressing the potential disparities in the application of AI/ML technologies [28,29]. While computers are often characterized as “unbiased machines”, they will most often perpetuate or amplify existing biases in the source data [30,31]. On the contrary, attempts to use AI to mitigate bias by identifying implicit partiality in clinical decision-making can yield positive results, but requires precaution and oversight to continually monitor the influence of bias [31]. If we ignore the issue with diversity at all levels, including those who are tasked with building the various AI technologies, we will further magnify the effect of problems created by implicit and explicit bias on these healthcare advancements.

### The digital health data divide

Commonly, clinical interventions have been designed for prevailing populations. Indeed, risk scores that account for race may over- or under-estimate risk assessments [32], with obvious consequences. Meanwhile, clinical diagnostics are defined for the few. As an example, the rarity of darker skin in dermatology can lead to the underdiagnosis of various diseases [33]. Considering that genetic data often does not include minorities, these populations are left underrepresented and, therefore, without access to personalized treatment or diagnostic tools [17,34].

Health outcomes are further complicated by the reality that certain clinical devices underperform at creating equitable assessments across race, sex, and other physical differences. For example, a study on pulse oximeter accuracy identified that darker skin overestimated arterial oxygen saturation, resulting in differences in treatment interventions [35]. Furthermore, a study on total hip arthroplasty outcomes documented that at one-year follow-up, women were more likely than men to report needing assistance in daily activities [36]. Additional data and research is necessary to account for genetic and physiological differences. Inaccurate technology development, which incorporates known and unknown biases, can have immense negative implications on diagnoses, treatments, and therapies implemented by clinicians and developers. As such findings continue to be revealed, the health gap in marginalized communities will only widen as they bear the burden of receiving treatments designed without proper adjustments for their unique community needs.

Health data is increasingly being generated at an astounding pace, with electronic health systems recording clinical notes and processes captured by clinicians and administrators at practically every stage of the care process. Data, such as those from monitors and imaging, is now automatically generated by devices and machines [7]. In recent years, the innovation of wearable devices and mobilized healthcare has provided the opportunity to collect large amounts of data outside of the hospital with the aim of implementing customized digital healthcare solutions. [37] These data are extremely valuable for learning and process improvement, especially when made openly available to clinicians and researchers [38].

While the expansion of health data is accelerating, the growth is not evenly distributed. The majority of available digital data are from wealthy regions with expanding adoption of electronic health records (EHR) and devices [39]. This is leading to a widening data divide, where large segments of populations, particularly the poor and those with low accessibility, are not captured digitally and are underrepresented in the resulting data sets [1].

The utility of AI/ML in healthcare seems boundless, but this area of data science must be approached with caution. Researchers tasked with collecting and interpreting data have an ethical responsibility to ensure that the development strategy for designing models and systems is both efficient and equitable.

### Objectives

This scoping review aims to investigate the landscape of existing research in the area of health data poverty. Our aim is to evaluate the current state of health data used in building AI models and the potential role it plays in exacerbating or alleviating existing disparities. We hope to assess potential exacerbating factors that contribute to data poverty, identify why these barriers exist, and recommend how they might be alleviated.

## Methods

A comprehensive search of the literature was constructed and performed by a qualified medical librarian (RSH). Medline (Ovid), Embase (Ovid), Scopus, and Google Scholar were queried using both natural language and controlled vocabulary terms for data poverty, digital health, artificial intelligence, vulnerable/underrepresented populations, bias, inequities, and health outcomes (S1 Appendix).

The results were then assessed and scored by the researchers on quantitative and qualitative measures. The assessment was completed by the group of authors, randomly assigned with crossover assignments. To reduce internal biases, every article was independently analyzed by at least two reviewers, and the final score was achieved based on consensus in discordant cases. We extracted relevant data, including 1) type of article, 2) publication year, 3) country where research was conducted, 4) nationality of primary authors, and 5) the study and author country income classification according to the World Bank classification [40]. Each study was then scored by the reviewers (on a 0–5 scale), attuning to subjective appropriateness to the topic of data poverty. In this review, we included the articles with reviewers’ scores of 3, 4, or 5.

## Results

**Fig 1:**
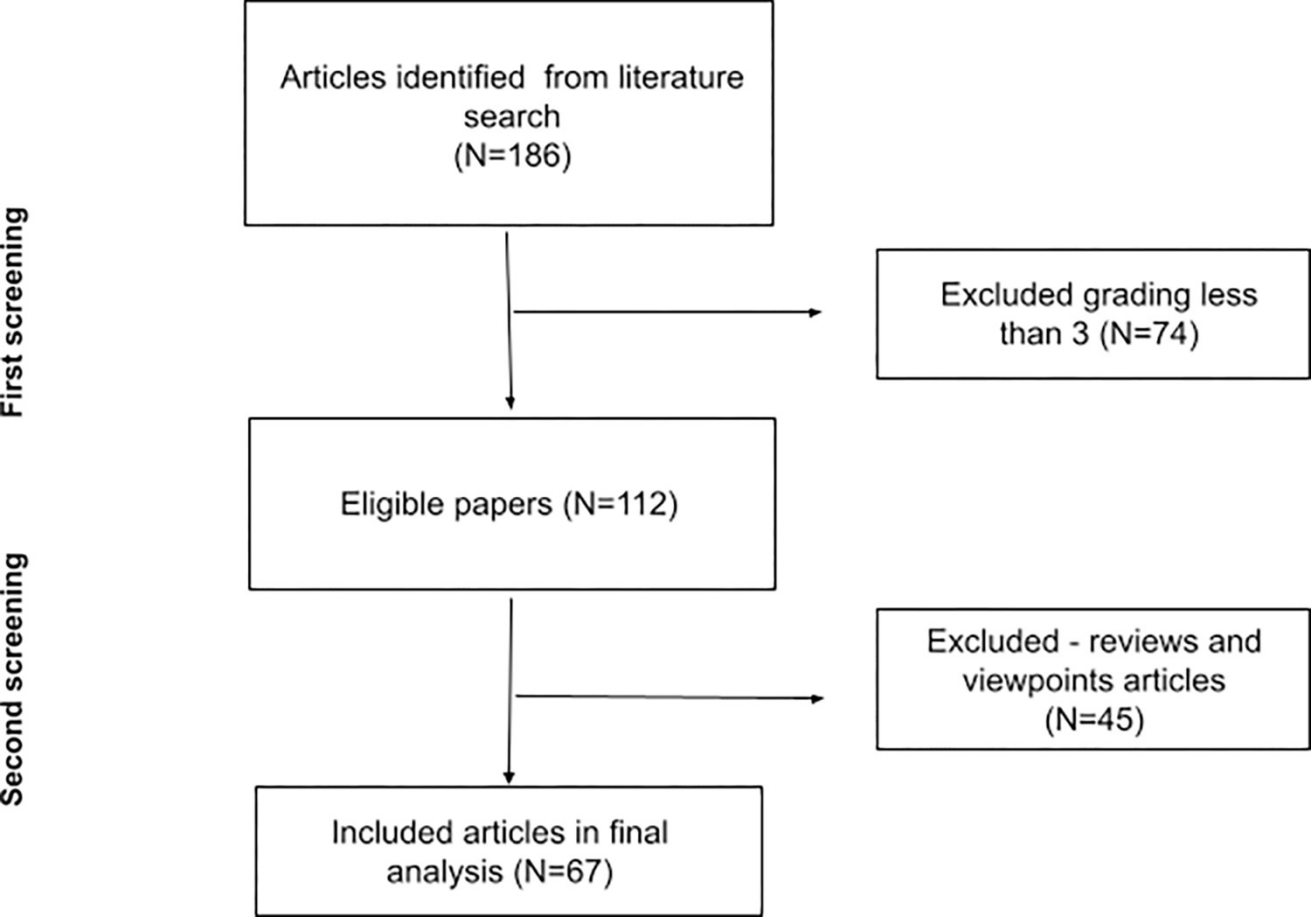
Flowchart of results.

While the topic of data poverty in healthcare is uncommon, our initial search produced a fair number (n = 186) of published papers. Our first screening filtered these papers based on adherence to data poverty: whether they directly acknowledged data poverty or indirectly addressed a cause or effect of AI-exacerbated biases. The 112 eligible papers underwent a second screening, where only original studies (n = 67) were considered for inclusion in the scoping review. Reviews and opinion papers were assessed and included for reference and discussion but excluded from the analysis of this review (Fig 1).

### Categories

To characterize the papers, reviewers assigned up to three relevant topic categories for each paper (Table 1). The main categories were:

- *AI/ML Bias—*These studies identified biases that were unintentionally perpetuated or amplified by AI algorithms.
- *Data bias—*These studies identified biases existing in the input data used to build models, such as missing data and its causes.
- *Disparities—*These studies looked at how various disparities, such as racial, socioeconomic, rural/urban, age, etc., were reflected in digital technology applications.
- *Population Selection—*These studies evaluated population selection, often by identifying under or over-representation of certain groups.
- *Clinical Outcomes—*These studies evaluated the direct effects and clinical outcomes of digital technology applications in healthcare.

**Table 1:** Results of categorization of studies.

Categories	# of Studies	Potential Subcategories
Disparities	48	Racial
		Rural/urban
		Socioeconomic/poverty
		Age/Sex/Gender
		Accessibility
AI/ML Bias	19	Algorithmic or modeling bias
		Errors in machine/deep learning
Data bias	12	Biased data source
		Missing data
		Transparency
		Patient mistrust
Population Selection	11	Representation
Clinical Outcomes	2	Errors diagnosis
		Errors in predicting health risks

Subcategories that detailed a particular aspect of the parent category were identified for most of the papers, but were deemed to not warrant a separate main category label. These were useful in highlighting the scope and diversity of the causes or impacts of the instigating factors.

The majority of the papers focused on disparities, which were usually racial or socioeconomic [41–45]. An important topic within this category is accessibility, which is more difficult to measure, and reflects the patient’s inability to access, utilize, or afford appropriate care. It also relates to physicians who serve communities that lack access to necessary equipment for AI/ML healthcare technology (e.g., rural communities) [44,46].

Data bias is the most straightforwardly related to data poverty. Biased data results in biased algorithms, which may be attenuated to a degree with careful tuning and continuous monitoring [18,47–52]. The sources of biased data are manifold. Most common is the disproportionate representation of wealthy or majority populations and the corresponding underrepresentation of minority populations [53]. Missing data can sometimes be accounted for in building AI models, however, this is only marginally effective when the missing data is similar to the existing data [47,51,52]. Missing data often reflects the issue of accessibility: poorer populations who do not have access to healthcare cannot contribute data. Lack of trust may also be a factor, where members of historically disadvantaged groups may be reluctant to share their data or participate in studies [47].

### Specialty

Where possible, the general clinical specialty was recorded and tallied (Table 2). In this review, the papers fell under thirteen specialties. Data science articles were the most prevalent, followed by social science and public health. These papers generally looked at registry data or general hospital data, without a specific medical specialty. Among medical specialties, there are radiology, oncology, genetics, ophthalmology, dermatology, and neurology articles.

**Table 2:** Tally of specialties discussed across studies.

All papers		First screening		Studies	
Data Science	72	Data Science	37	Data Science	24
Social Sciences	31	Social Sciences	22	Social Sciences	17
Public health	20	Public health	13	Public health	6
Other	13	Other	8	Other	5
Radiology	10	Radiology	5	Radiology	5
Dermatology	9	Dermatology	6	Dermatology	0
Oncology	6	Oncology	5	Oncology	4
Genetics	6	Genetics	4	Genetics	3
Ophthalmology	5	Ophthalmology	3	Ophthalmology	2
Neurology	3	Neurology	3	Neurology	1
Policy	2	Policy	1	Policy	0
Basic Sciences	2	Basic Sciences	0	Basic Sciences	0
Psychology	1	Psychology	0	Psychology	0
Cardiology	1	Cardiology	1	Cardiology	0

Papers on data science primarily focused on problems in AI/ML-related data, and how the digital determinants of health lead to algorithm problems at large, rather than contextualizing within a specific specialty. These papers often focused on primary care medicine or disease-specific (e.g., COVID-19) analyses across specialties [34,54,55]. For example, data bias in EHR data may lead to misclassifications and less accurate predictions for select groups [56]. Proposed solutions were varied. One suggestion was to adjust the models: the Joint Fairness Model is a logistic regression model that estimates group-specific classifiers that incorporate fairness for prediction [57]. Addressing other steps within the development process, such as creating inclusive data standards to support interoperability, data and code sharing, and determining AI reliability through development metrics, may also be helpful [58].

The social science papers focused on sociocultural factors that contributed to data disparities. Disparities in race, socioeconomics, and internet access lead to data poverty. Often, there are limited and/or missing race and ethnicity classifications, such as Native Hawaiians and Pacific Islanders. This is a form of structural racism, as failure to identify race or ethnicity may hide the social determinants of health for these populations [42,47]. The countervailing challenge is that merely identifying the race or ethnic data may lead to biases in treatment as well, so there is no clear solution [59]. We need complete representative data when building the models, but we must also address the ingrained or unconscious biases at bedside. Access to treatment itself is complicated by poverty. Moreover, unequal internet access, and therefore unequal digital health access, is often experienced by the elderly [60], certain global regions [61], and rural areas [62].

In data poverty articles that discussed genetics, analyses focused on how underrepresentation of populations leads to diagnostic mistakes and inappropriate pharmacological treatments. Disparities in genetics are seen in the unbalanced distribution of genomic data in various populations. For example, African, Polynesian, and Brazilian genomic data remain underrepresented or even ignored, despite the significant contributions this data can make in advancing our understanding of the human genome [16,17,58,63]. These genetic variations matter and must be represented in data. The current method of recording racial/ethnic/genetic data is grossly deficient.

In radiology, AI can be used to enhance diagnosis and follow-up. Yet, the efficacy of algorithms varies across situations. Bias in imaging can occur due to machine-induced variance [64]. A study by Dhont et al. applied five neural networks to recognize COVID-19 pneumonia through chest radiography, but the algorithm recognized the site (e.g., hospital, clinic) where the radiograph was done and not the disease itself. When the model was trained on a reliable and realistic single-source dataset, the sensitivity results were low, at less than 70% [55]. These studies highlight the challenges of generalizing findings between devices, clinicians, and institutions.

It was a repeated theme across specialties that disparities in outcomes were often driven by applying models despite having underrepresented populations in the data set. Consequently, disparities have been identified across specialties. Cancer treatment outcomes have been observed due to inappropriate risk assessment and, therefore, inappropriate preventive practices [65]. In ophthalmology articles, there was an identified lack of representation of various demographic characteristics and pathological entities in publicly available datasets, prompting the need for a collaborative approach to reach real-world deployment [15,66,67].

### Represented countries

The distribution of the country of study and country of the authors was relatively comparable (Table 3). The most common country of study was the United States of America, followed by papers that included multiple countries. Most of the studied countries were developed countries, where the multiple country papers often attempted to compare and contrast countries in different economic states. For even the wealthy countries, studies still noted disparities across populations, likely driven by factors such as socioeconomic, racial differences, or a rural/urban divide [62]. Notably, the only low- and middle-income countries (LMIC) independently studied in our review were China, India, and Lebanon [18,68,69]. There were no participant countries nor authors from the African continent. Importantly, the challenges in mentorship were evident. Authors were mostly from high-income countries, and authors from LMICs were rarely first authors.

**Table 3:** Representation of countries studied and author nationality.

Country	Study	Author
US	30	31
Multiple Countries	13	15
None/Unknown	3	0
Australia	3	3
Germany	3	2
UK	3	3
Canada	2	2
Spain	2	3
China	1	1
Finland	1	1
Greece	1	1
India	1	1
Israel	1	1
Lebanon	1	1
Netherland	1	1
Poland	1	1

## Discussion

### Assessment

Improving health care itself is an immensely multifaceted problem. When compounded by highly technical digital technologies, the variables and outcomes are exponentially more difficult to monitor. In this review, we discuss how health data poverty is a complicated problem without a straightforward solution. Disparities can infiltrate anywhere along the application development process. Disparities are also inherently a systemic problem, where existing biases will propagate even if the tools and processes are unbiased.

Proper evaluation of complicated issues is essential to avoid further pervading disparities that exist in accessibility to the data. It is necessary for data scientists, researchers, and clinicians to account for struggles with injustice, data selection, and the application of the tools made as a result of the data. Furthermore, access to big data illuminates one of the most important ethical questions in health data poverty: who benefits? Although big data can have extensive value, irresponsible development can be dangerous. Organizations in possession of these large data sets can choose to provide free access to the information to improve society, or they contrarily use it to further the company’s financial gain [70]. The accessibility, application, and distribution of health data sets require stricter oversight and regulation, to ensure they benefit the social good. The ideal would be that the data most benefits those like the patients who contributed to the data, rather than only the institutions that collected the data.

### Essential problems with the tools and data

**Fig 2:**
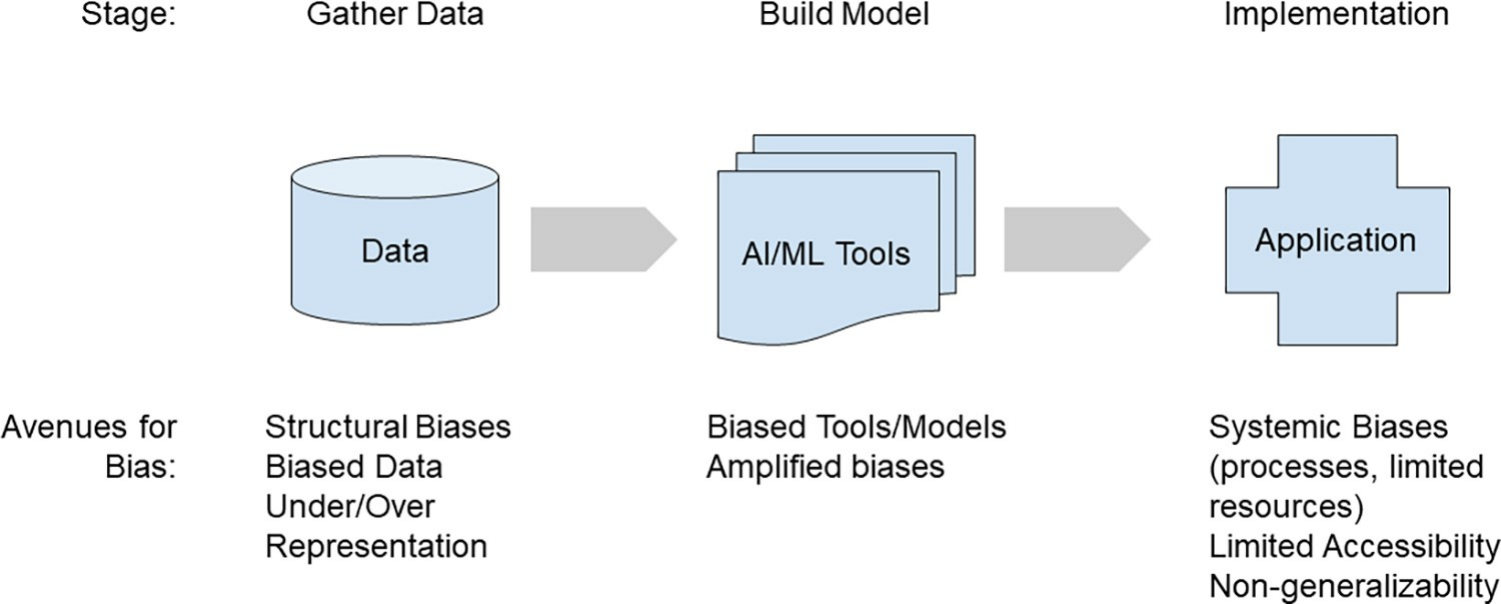
Potential avenues of bias in the Health AI development process.

The AI/ML development process can be simplified into three stages: (1) Data, (2) Model, and (3) Implementation (Fig 2). Biases can be introduced at any stage of this process; the authors denote these agents of bias as source, amplifier, or interaction. Firstly, the source, which is the data or tool itself, may inherently introduce bias into the entirety of the development process due to its own deficiencies. This bias perpetuates, even if every subsequent step is unbiased. Additionally, data might appear unbiased, potentially due to insufficient sample size or unbalanced representation. Secondly, an amplifier may magnify a modest bias which becomes more apparent after a tool is applied. Lastly, the interaction of standalone aspects, which initially appear unbiased, may result in unforeseen processes that introduce bias. Importantly, the effects of these agents may not appear until later stages of the development process, nor equally across implementation.

Increased computational power and data availability have driven the growth of ML in healthcare over the last decade. However, problems like missing data sets for certain subgroups impact the ability to generalize the datasets and the AI algorithms created [58]. Flawed algorithms mean the creation of defective models used to operate machines, further amplifying systemic underrepresentation of different populations. For example, when applying the Framingham Risk Score to populations with similar clinical characteristics, the predicted risk of a cardiovascular event was 20% lower for black individuals compared with white individuals, suggesting that the score may not adequately capture risk factors for some minority groups [30], and technologies developed and validated using these datasets, are not generalizable to the wider populations, such as children, ethnic minority groups, older adults and patients with disabilities. If an AI algorithm trained exclusively with U.S. data were used to predict the mortality of a Filipino COVID-19 population, predictions might be inaccurate and will be disadvantageous to populations not represented in the large datasets commonly used to build these models. This can both reinforce existing health inequities and cause possible harm amongst minority patients, giving rise to other associated ethical issues. Instead of narrowing the health gap, such technologies instead widen the digital divide through the health data poverty borne out of asymmetrical datasets. Consequently, underrepresented people might be unable to benefit from these data-driven interventions and could even be harmed by them.

The increased use of digital technologies also creates a potential for biased datasets. Inadequate access to the internet and other essential technology is a structural problem that affects health, education, and the economy, contributing to data poverty. Datasets from pervasive sensing, mobile technologies, and social media can under-represent or exclude those without digital access. Urban and rural discrepancies in internet access significantly contribute to the disparity in data generation and access to digital healthcare solutions. The unwillingness to use the internet and share data due to concern about confidentiality breaches, data leakage, and commercial use or abuse of data also contributes to data poverty (46).

### Takeaways

While advancements in AI/ML have the potential to improve healthcare, we must monitor their development with strict caution. The basis for this argument is that AI/ML is limited by the quality of the data used to program the technology [31]. One unrealistic perspective is that AI can help remove bias from fields like healthcare by creating standardized testing and outcomes, irrespective of a physician’s explicit or implicit bias. However, the undetected structural biases are perpetuated even with unbiased clinicians and tools. Furthermore, bias during the selection process for data sets chosen for algorithms can be detrimental if it fails to assess crucial factors like race, gender, or ethnicity, leading to algorithms absent internal and external validity.

Addressing these concerns requires a collaborative approach among various stakeholders towards a common goal. Although advancements in AI/ML may differ between countries, a standardized approach should ensure that data are available for the benefit of the population from which they were collected, with the opportunity for ongoing development and testing of digital health technologies that will improve the health of that population. Prioritization of datasets will also vary by country, but important features that should be considered include contextualized local health needs, appropriateness of specific digital health solutions, and the facility and resources needed to support that digital health solution.

#### Data representation matters

In AI/ML, using health data not inclusive of various populations leads to inaccurate generalizations for digital health [1]. Continuing to create technologies based on these incomplete datasets can be inappropriate and even dangerous [59]. When data from non-representative and biased datasets are used to encode machine learning and deep learning, the resulting algorithms may be biased, further compounding existing inequalities in health care and research [34,55,71,72]. Improvement in the collection of inclusive data from a variety of different populations (e.g., sex, race, gender, ethnicity) results in algorithms that are appropriately designed for a wider population while reducing biases [73].

The data used to make and validate AI models often under-represent the general population. This lack of variation in datasets is known to amplify biases in a population, particularly for minoritized subgroups. Cheng et al. highlight that models can have disparate impacts on discriminated subgroups even when the real dataset is not directly used in the downstream training process and even when the synthetic dataset used for the training is balanced [74]. For instance, the use of biased data in facial recognition algorithms has resulted in poorer recognition rates for black female faces in commercially deployed algorithms [75]. Generated, but biased, data will only exacerbate the prevailing underrepresented population whether through class imbalance or having small minority sample sizes in the underlying training dataset. Furthermore, generating synthetic data to replace dataset struggles to capture the proportions that exist in the real data and fails to reduce fairness concerns for subgroups of any given attribute, potentially leading to changed representation, and introducing bias.

The prevalence and incidence of diseases and their risk factors often vary by population group. If the data do not adequately represent the population at risk, then models used in AI/ML might have varying metrics, leading to suboptimal results and possible harm to underrepresented minorities. The importance of these data representations must become a core principle at the outset of technology design and not an afterthought as it currently is with many tools.

As such, data limitations are an important entity that can result in bias. These sources of bias in AI/ML may present, in most, if not all stages of the development process of the algorithm. It is crucial to address these biases that may propagate unknowingly since data are used to create upstream embeddings that facilitate downstream transfer tasks. Dullerud et al. elucidate how imbalanced data significantly affected downstream classifications even with balanced training data, suggesting that data cannot be used to address downstream classifiers from imbalanced beddings [76].

### Recommendations

#### Be aware of the limitations of AI/ML tools

As AI/ML tools become more advanced, they become more out of the bounds of typical human understanding. This is already evident with the growing use of unsupervised learning and neural networks, where AI/ML algorithms are treated as a simple black box and some idly trust the results [77]. There is a push for explainable AI, where the tools attempt to highlight the reason or features that contribute to a particular prediction, however this is not universally utilized and ignores potential interactions when multiple tools are utilized [78,79]. Clinicians and researchers must be aware of the limitations of the AI/ML tools they deploy and understand the implications based along the entire development process. From generating the input source data, all the way through implementation, there are myriad elements that can negatively impact outcomes. Differing patient populations, clinician resources, background clinical training, or even equipment can result in widely varied results. All of these and their interactions need to be well understood in order to adjust the deployed tools for equity.

#### Generate equitable data

In order for these AI models to be inclusive, the data used need to not only be accurate but also representative of the needs of diverse populations. Implementing continuous monitoring and transparency to measure impacts of biases of AI/ML design and evaluation tools could help strengthen collaborations between the AI and medical fields, and open up the space for various entities to participate in AI deployment for medicine. Safety assurance will be needed before deployment of AI systems in the healthcare setting with continuous monitoring and collection of data and experiences from the use of these systems. In addition, strong measures need to be adopted from public authorities to ensure security and avoid abuse of data. Collective enforcement in the data protection domain should be enabled and facilitated. This review summarizes the complex, multifaceted problem of health data poverty and the need for collaborative efforts to end it.

Representativeness in AI/ML should be the main focus in datasets and algorithms development to minimize the risk of the perpetuation of unequal digital healthcare. While data sources mainly come from privileged populations in a few high-income countries, the inequalities in digital solutions will remain, increasing the digital divide and contributing to the disconnect in the adoption of technologies. As data is collected, we must be aware of and avoid biases. The representative inclusion of every race and sociodemographic group is essential in a healthcare dataset to avoid inequitable algorithm performance.

We must build trust by assuring data confidentiality and security. Data generation, collection, and sharing should be endorsed between institutions and among all medical specialties to increase data representativeness and ML/AI fairness. To caution, there is a heightened ethical challenge when collecting race/ethnicity-based data as these are highly susceptible to misuse or abuse. However, these information and associations are also necessary to analyze and uncover unseen or inherent systemic biases. Rigorous regulation, competent management, and continued oversight is required to protect those populations affected by the implementation of these systems.

Ultimately, rather than only researching and advancing the development of AI/ML tools, continued monitoring after deployment focusing on clinical outcomes will be essential. The downstream analysis of the resultant outcomes will determine which factors need to be prioritized for rebalancing. To accomplish this, the full stream of input data, open-sourced algorithmic models, and outcome data need to be made available and studied extensively.

### Limitations

Our study has several limitations. Firstly, our analysis was limited to articles published in English, Spanish, and Portuguese, excluding articles in other languages. Secondly, the assessment of scores relied on subjective judgments, which introduces the possibility of internal biases. We attempted to alleviate some of these by convening a diverse group of authors from various disciplines and geographic locations and the utilization of independent graders for scoring, reducing the potential effects associated with subjective and internal biases.

## Conclusion

Because health data inherently influences the output of AI/ML, transformative efforts must be focused on the refinement of the pre-selection process for datasets while continuing to monitor the technology throughout its development. Digital solutions have the potential to improve healthcare quality and delivery, but the awareness of health data poverty as a digital determinant of health is necessary to assure fairness and representativeness [80]. With the increasing use of AI/ML in healthcare, the potential for health inequities it poses must be addressed. AI/ML systems have complex cycles, involving data acquisition, training, development, and recalibration, thus, requiring a multidisciplinary approach. This will allow for dedicated efforts to address their impact and advise organizations, regulatory bodies, health systems, and governments for technology that is more digitally inclusive.

## Supporting information

S1 PRISMA Checklist PRISMA checklist. (PDF)

S1 Appendix Search strategy. (DOCX)
