# Supplementary material for: Digital Determinants of Health: Health data poverty amplifies existing health disparities—A scoping review
Source: PLOS Digit Health. 2023 Oct 12;2(10):e0000313. doi: 10.1371/journal.pdig.0000313 (PMC10569513; doi:10.1371/journal.pdig.0000313)
Supplement: S1 Appendix — (DOCX) [file pdig.0000313.s002.docx]

##### S1 Appendix: Search Strategy

| 1. | "data poverty".ab,kf,ti. |
| --- | --- |
| 2. | "data poor".ab,kf,ti. |
| 3. | ("underrepresent*" adj5 data*).ab,kf,ti. |
| 4. | (data* adj2 (absent* or scarc* or inaccur*)).ab,kf,ti. |
| 5. | or/1-4 |
| 6. | dataset/ |
| 7. | "Datasets as Topic"/ |
| 8. | (data or dataset*).ti,kf. |
| 9. | or/6-8 |
| 10. | (poverty or disparit* or equity or inequit* or inequalit* or equality or transpar* or bias or prejudic* or underrepresent* or underserv* or poor or disadvantag* or "digital divide" or "lack of representation" or vulnerable or Scarcity or scare or inaccur* or litera* or illitera*).ab,kf,ti. /freq=3 |
| 11. | 9 and 10 |
| 12. | 5 or 11 [data poverty] |
| 13. | exp *Artificial Intelligence/ or exp *Telemedicine/ |
| 14. | exp *Pattern Recognition, Automated/ or exp *Mobile Applications/ |
| 15. | exp *Machine Learning/ or exp Wearable Electronic Devices/ or exp *Data Mining/ or exp *Big Data/ or exp *Computational Biology/ |
| 16. | exp *Diagnosis, Computer-Assisted/ or exp digital divide/ |
| 17. | ("artificial intelligence" or "machine-learning" or "digital" or algorithm* or ehealth or mhealth or telehealth* or telemedicine).ab,kf,ti. /freq=2 |
| 18. | or/13-17 [Digital terms] |
| 19. | 12 and 18 [data poverty and digital] |
